# Implementation and Sustainability of Hospital-based Addiction Consultation Services

**DOI:** 10.21203/rs.3.rs-8177037/v1

**Published:** 2025-12-15

**Authors:** Sandra K. Evans, Allison J. Ober, Allyson Gittens, Alex Peltz, Itai Danovitch

**Affiliations:** RAND; Ober Insights; BME Strategies; RAND; Cedars-Sinai Medical Center

**Keywords:** Addiction Consultation Services (ACS), Opioid Use Disorder (OUD), Medication for Opioid Use Disorder (MOUD), Substance Use Disorder (SUD), Hospital-based Care, Sustainability, Program Sustainability Assessment Tool (PSAT), Treatment Linkage, Implementation Barriers

## Abstract

**Background:**

Substance use disorder (SUD) is prevalent among hospitalized patients, yet most acute care settings do not routinely provide evidence-based SUD treatment. Hospital-based addiction consultation services (ACSs) have emerged as a promising strategy to increase the use of medications for SUD and improve linkage to post-discharge care. However, despite evidence supporting ACS effectiveness, these programs remain difficult to sustain, and their growth has been concentrated in a relatively small minority of hospitals. Understanding the factors that enable ACS sustainment and the barriers programs encounter is critical to expanding evidence-based SUD treatment across the broader hospital landscape.

**Methods:**

We conducted semi-structured interviews followed by surveys with 21 leaders and representatives of ACSs at 17 hospitals across the United States. Participants were identified through purposive and snowball sampling via addiction medicine professional networks and correspondence. Interviews explored reasons for ACS initiation, configuration, barriers, and factors supporting sustainment, guided by constructs from the Program Sustainability Assessment Tool (PSAT), including environmental support, funding stability, organizational capacity, strategic planning, partnerships, communications, monitoring, and evaluation. Survey items based on the PSAT asked participants to rate the extent to which their hospital had engaged in various activities related to implementation and sustainment of the ACS. We describe common themes based on qualitative coding of interview data and the analysis of median scores and interquartile ranges for each relevant PSAT domain.

**Conclusion:**

Findings show that funding stability remains the lowest scored factor for ACS sustainability, with fee-for-service models and lack of adequate post-discharge treatment services as ongoing barriers. Workforce issues, hospital department friction, and external partnership challenges were also noted. Most ACSs are supported by diversified funding and cross-disciplinary staffing. High median survey scores in most domains reflect strong support for sustainment, but addressing ongoing barriers is crucial for long-term success of ACSs in improving care for hospital inpatients with SUDs.

**Trial Registration:**

Clinical trial number: not applicable

## Introduction

In 2024, 16.8% of the U.S. population (48.4 million people) had a substance use disorder (SUD), including 27.9 million people who had an alcohol use disorder, 4.9 million who had an opioid use disorder (OUD), and 28.2 million people who had any drug use disorder [1]. People with SUDs frequently present to acute care settings with direct and indirect consequences of their substance use, and experience markedly worse outcomes than the general population [2]. People with an SUD have a 24% greater likelihood of an unplanned readmission to the hospital [3] and people with OUD are four times more likely to have a fatal overdose in the first two days after hospital discharge [4] indicating the need for additional support both during and after the hospital stay.

Addiction consultation services (ACSs) can improve the implementation of evidence-based treatments (such as medications for alcohol or opioid use disorder) during acute hospitalization and facilitate post-discharge treatment engagement for people with SUD [5–7]. For people with OUD in particular, the inpatient setting represents a pivotal touchpoint where patients can initiate life-saving medications for OUD and receive facilitated linkage to follow-up care in the community.

Results from the Substance Use Treatment and Recovery Team (START) Randomized Clinical Trial (RCT) showed that in comparison to usual care participants, START participants were significantly more likely to initiate MOUD in the hospital, have a discharge plan with an OUD focus, and initiate or continue MOUD after being discharged [5]. Other studies such as a pilot RCT of START, as well as a stepped-wedge trial and multiple observational studies also have demonstrated that ACSs improve linkage to MOUD and post-discharge care, and have found ACS implementation to be associated with reduced rates of patient-directed discharges, reduced in-hospital stigma, improved patient trust in hospital clinicians, and an overall reduction in post-discharge mortality [6–10].

ACSs are generally comprised of an interprofessional team capable of delivering MOUD and supportive services [11]. The most common roles are a physician to medically manage SUD and a care manager to deliver brief interventions and coordinate linkage to aftercare [5, 8, 11]. Other roles and ACS activities include social workers to provide psychosocial assessments and assist with care planning, nurses to help administer medication and monitor withdrawal, pharmacists to guide safe medication use, and peer recovery coaches to offer lived-experience support.

ACS teams also frequently provide education and support to hospital staff to improve competence in SUD management, reduce stigma, and model best practices for addiction treatment. Activities can include withdrawal management, pain management, harm reduction counseling, and direct coordination of transitions of care to outpatient SUD treatment or community-based resources, as well as follow-up phone calls. The composition and focus of ACS teams vary according to institutional needs and resources [8]. Team composition and department location vary, and specific placement depends on institutional resources, size, and priorities, though an addiction medicine or psychiatry physician is generally considered essential [8].

Despite growing evidence supporting the effectiveness of ACSs, there is limited information on their implementation and sustainment, with just one article to date (Priest et al., 2020) addressing implementation issues [12]. Based on interviews with addiction physicians at 16 U.S. hospitals involved with or planning ACSs, Priest et al. suggest that barriers to the development and operations of ACSs include stigma and discrimination from both hospital administration and external stakeholders, limited addiction-related resources, challenges with access to community-based treatment programs, restrictive MOUD policies, and challenges stemming from low-reimbursement models [12]. There remains a substantial gap in the literature regarding factors that support initial implementation and long-term sustainability, financing, and scale-up of ACS programs across diverse hospital environments. This information is critically needed to advance policy support and funding for, and dissemination of, ACS in hospitals with varied resources and capabilities.

To address this gap, we conducted a mixed-methods study using web-based surveys and semi-structured qualitative interviews with 21 addiction medicine professionals working in ACSs in 17 hospitals across the U.S. to understand reasons ACSs were initiated, how they were initially configured and have evolved over time, and key factors supporting their initiation and sustainment. In this article, we present findings from this work and offer considerations for hospitals interested in implementing and sustaining an ACS to improve evidence-based care for hospitalized patients with OUD.

## Methods

We used a combination of semi-structured interviews and follow-up surveys with interviewees to gather insights from leaders and representatives of ACS at hospitals throughout the U.S. In interviews, researchers asked interviewees about the story of each ACS including how it started and how it has been sustained over time.

Our survey and qualitative work was guided by key sustainability constructs highlighted in the Program Sustainability Assessment Tool (PSAT) [13]. The PSAT was developed to guide assessment of capacity for sustainment of public health programs.

### Participants

We used a purposive sampling approach to select participants. Criteria for selection included a focus on leaders of ACS in hospitals around the U.S. Specifically, we sought out hospital administrators or other staff who were knowledgeable about the implementation of an ACS in a hospital setting.

We contacted potential participants through mass emails to widely used listservs including Academy of Consultation Liaison Psychiatry Addiction Consult Service interest group, and the American Society of Addiction Medicine listserv of hospital addiction medicine specialists. We followed up to schedule interviews with those who responded. We also used snowball sampling to locate additional representatives of ACSs. Finally, we developed a list of names of those affiliated with ACSs based on academic journal publications. In some instances, we cold-emailed contacts from that list. In other instances the names on that list overlapped with contacts from listserv emails. We conducted interviews until we reached the saturation point when all three researchers agreed that very similar topics and themes were emerging from each interview. Interviewees were asked to complete a follow-up survey after each interview was completed.

### Procedures

We conducted semi-structured interviews with 21 individuals representing ACS at 17 hospital sites. These interviews took place between August and December 2024. Interviews lasted between 40–60 minutes and conducted virtually via Microsoft Teams. Audio from interviews was transcribed to facilitate analysis.

We developed a semi-structured interview guide consisting of open-ended questions designed around PSAT sustainability factors including environmental support, funding and funding sustainability, organizational capacity, program evaluation and adaptation, communications, strategic planning and implementation strategies, and partnerships (within and out the hospital setting). The guide also included questions on barriers related to implementing and sustaining the ACS (see Table 1).

After each interview, we emailed a link to an online questionnaire to each participant. Survey items asked participants to rate several factors related to the ACS: environmental support, funding stability, strategic planning and organizational capacity, the extent of monitoring and evaluation, and external partnerships.

**Figure 1:**
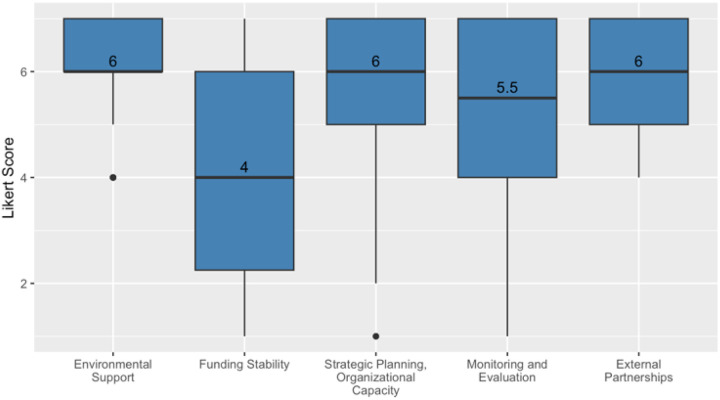
Median and IQR results by PSAT domains: environmental support, funding stability, strategic planning and organizational capacity, monitoring and evaluation, and external partnerships.

Survey responses were aggregated at the domain level (see Figure 1) by combining scores from each category. The reliability of these combined scores was assessed using Cronbach’s alpha, with the following results: 0.79 for environmental support, 0.83 for funding stability, not scored for external partnerships, as there was only one question, 0.90 for monitoring and evaluation, and 0.79 for strategic planning and organizational capacity. Strategic planning and organizational capacity were consolidated into a single domain due to highly correlated survey items as both domains consider the roles and abilities of ACS staff. Participants rated statements in each domain on a scale from 1 to 7, indicating the extent to which the hospital had done the stated activity, toward implementation and sustainment of the ACS, with 1 indicating “no extent” and 7 indicating “to a very great extent.”

By using both interviews and surveys, this study was able to collect comprehensive data about each ACS based on a limited number of interviews. Participants were offered $100 in the form of a gift card as an incentive to complete the interview and questionnaire.

### Qualitative Interview Data Analysis

To analyze interview data, we used an inductive and deductive approach. First, we developed a codebook derived from the question topics in the interview guide in PSAT categories (see Table 2). After reading all transcripts, we developed codes around emergent themes. Three researchers then practice-coded and discussed the codebook at length to refine and finalize the codebook. After the codebook was finalized, the researchers completed an inter-rater reliability assessment.

To assess inter-rater reliability, the coding team (the PI and two team members) conducted three rounds of coding. First, they coded the same transcript to help refine the codebook and add to the code definitions. Next, each researcher coded a unique transcript within Dedoose, a qualitative analysis software program [14]. All coded excerpts were then exported to Excel; the two other coders who had not coded in Dedoose then blind-coded the excerpts in Excel. The purpose of this exercise was to test the extent to which coders applied the same codes to each excerpt across three different transcripts. The results were three transcripts triple-coded by the three researchers. Next, we conducted an IRR test (Krippendorff’s alpha). The results were lower than optimal for intercoder reliability, so we reviewed discrepancies and clarified codes in the codebook. We then repeated the assessment with a revised codebook. The Krippendorff’s alpha test on main (parent) codes equaled 0.704 (95% CI: 0.582, 0.813), which is considered acceptable [15].

### Survey Data Analysis

We summarized central tendencies using median scores and interquartile ranges for each relevant PSAT domain (Environmental Support, Funding, Organizational Capacity, Partnerships, Adaptability and Strategic Planning, Communications, and Program Evaluation). We created box-and-whisker plots for each domain, using the ggplot2 package in R [16]. Each box-and-whisker plot represents the distribution of the score for each statement for all respondents across a range from 1 to 7. Each box shows scores from the 25th to the 75th percentile, with the median shown as a line across the middle of the box. The whiskers extend from the box to the smallest and largest scores that are not considered outliers. Scores outside these fences (the lower fence is set at 1.5 times the interquartile range below the 25th percentile, and the upper fence is 1.5 times the interquartile range above the 75th percentile) are considered outliers and are shown as individual dots.

## Results

### Participant Characteristics

Of the 21 interviewees, 18 (86%) responded to the brief demographic and sustainability survey. Of these, most (83%) were physicians, female (61%), and White (83%); 22% were Hispanic or Latino. The majority (89%) of interviewees who responded to the survey were currently working as a clinician on the ACS and most (61%) ACSs had been implemented for longer than five years (see Table 3 for additional demographic data).

### About Participating ACSs

Participating ACSs (N=17) were located across four major geographic regions in the United States, with seven in the Northeast, five in the West, three in the Midwest, and one in the South. Most ACSs were established within the past decade, with six sites reporting a tenure of 10 or more years, seven reporting 5–9 years, and four reporting 1–4 years since inception. With respect to placement within the hospital, ACSs mostly resided within Psychiatry (n = 6) or Addiction Medicine departments (n = 4), while others were embedded in general medicine (n = 2), hospital medicine (n = 1), or internal/family medicine (n = 2); one hospital noted a combined psychiatry/VA placement.

Most hospitals (n = 10) utilized an interprofessional addiction consultation service model [11]. Another five sites described a psychiatry consultation-liaison service model, while two sites reported practice-based or individual consultant approaches.[1] ACS cross-departmental staffing included various combinations of addiction medicine physicians, nurse practitioners, social workers, and frequently incorporated medical trainees (fellows, residents, medical students), peer recovery coaches, and, in some programs, addiction counselors, care managers, patient navigators, or researchers. Initial program funding was most commonly derived from hospital or health system sources (n = 10), followed by grant funding (n = 7) and, to a lesser extent, government (non-grant), philanthropic/donor, volunteer/unfunded, or other sources. Current funding was diversified across hospital/health system support (n = 13), professional billing revenue (n = 3), grant (n = 5), and government (non-grant) funding streams (n = 4; see Table 4).

### PSAT Domain Themes

In this section, we present survey findings and themes that emerged from interviews regarding the factors participants considered to be important to initiating and sustaining ACSs over time. Findings are organized according to the PSAT categories: Environmental Support, Funding, Organizational Capacity, Partnerships, Adaptability and Strategic Planning, Communications, and Program Evaluation.

Overall, median survey scores within PSAT domains were high, with most above 5 (on a scale from 1 to 7), indicating fairly strong support of each factor toward sustainability of the ACS. The funding stability score was lowest. Figure 1 provides an overview of survey responses in each PSAT domain.

Table 5 provides an overview of themes from the qualitative interviews within PSAT domains. We describe interview findings and survey responses in greater detail below.

#### PSAT Domain: Environmental Support (Including internal partnerships)

**Figure 2:**
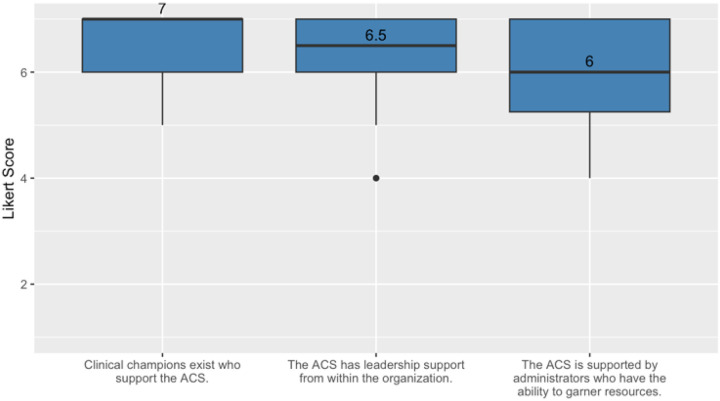
Median and IQR results for survey items assessing environmental support.

Most participants indicated in their surveys that environmental support was a key factor in initiating and sustaining the ACS. Half or more than half of respondents very strongly agreed with statements about having clinical champions and leadership support, with slightly less agreement for the ACS being supported by administrators who have access to resources (see Figure 2). Survey findings were also supported by themes derived from qualitative interviews

#### Theme: Widespread stakeholder-buy-in for initiating the ACS was common at most hospitals due to consensus around the imminent need for OUD treatment among inpatients.

Most participants said that there was clear recognition of the clinical need for OUD treatment in their hospital and discussed high rates of self-directed discharge as a consequence of untreated OUD. Many participants described how the benefits of an ACS were obvious to a wide range of hospital stakeholders, which led to widespread buy-in and support for initiating the ACS.

“I think at [hospital], there was a recognition that patients with substance use disorders did not have good hospital stays, had a high rate of self-directed discharge and that it was a source of frustration both for the patient and for the hospital teams taking care of them who felt like they had no tools to offer these patients support or help them through their stays, and we’re tired of seeing them come in and out. So the hospital made it a priority and provided funding initially.”

(ACS 12)

Agreement among hospital stakeholders that MOUD can effectively address OUD also contributed to the perception that ACSs could be used to better address the needs of patients with substance use disorders in the hospital setting.

“When I went back and did my Addiction Medicine Fellowship, it was very clear to me that hospitalists are like, we understand the urgency of getting people out discharged from the hospital, that’s such a big factor. And we understand how systems work and we do a lot of general medicine. And with sort of the movement of MOUD in this space and the fentanyl epidemic, it just made sense that hospitals could easily and efficiently do this work because we’re not doing a ton of behavioral interventions in the hospital. We’re managing their withdrawal and we’re getting them out of medicine and we’re figuring out where the next place is to receive dedicated addiction therapy and medications. So my thought has been, let’s get hospitalists to do this work as part of their routine practice.”

(ACS 6)

#### Theme: Leadership support and having “champions” throughout the hospital were critical to both the initiation and sustainment of ACSs.

The presence of internal champions (i.e., vocal advocates of addiction treatment and ACSs), sometimes across departments (e.g., hospital social work, leadership), was necessary not only for driving the initiation process but also continues to be needed for sustaining the ACS.

Advocacy and persistence were critical to getting most ACSs off the ground. Champions helped obtain buy-in by building internal coalitions and partnerships to help ACS function, for example by including medical fellows or residents, and in some cases, a mix of medical and psychiatric physicians to staff the ACS. Many champions also had to communicate the “business case” for the ACS to leadership.

“We [psychiatry] sort of underestimated how much we would have to get buy-in from [medicine and surgery]. Like we felt, in behavioral health and in psychiatry, it’s like very easy to be like, oh yeah, substance use, we could treat it, whatever. But then the buy in from medicine was it just took a bit of time…. when we were implementing or preparing for implementation, we had meetings like once a month to just talk about like where are we at with staffing? What are we doing with these workflows? And we would often invite people from medicine or surgery there….It almost feels now like if you took the service away, I feel like medicine would be like mad about it because they rely on them to help them out so that they can deal with all the other stuff they need to deal with.”

(ACS 15)

Local hospital leadership support and advocacy also were necessary for acquiring funding to launch the ACSs. Key leaders included departmental or division leadership (typically the division in which the ACS is housed and often resourced to some extent) as well as hospital administrators who oversee hospital services. These champions often took on additional work to build clinical and administrative support.

“Hospital administration buy-in is critical because we’re not a service that’s revenue generating. So somebody has to understand the value of this and agree to fund it. So I think you really need to have the administration that sees the value.”

(ACS 02)

Some participants described how it was most effective to show how the ACS aligned with top leaders’ priorities. That type of priority-driven connection made leadership support easier to obtain despite the fact that many ACSs were incurring expenses rather than generating profits for the hospital. Champions also helped initiate outreach within hospitals through meetings, educational sessions, and informational materials.

“I think that was definitely important of like having an advocate that was sitting in the admin place and could access the data and really show the need and show the work that was being done. I think that was like instrumental. And so, like my supervisor saw the value because he’s a psychiatrist and works in behavioral health. So it’s very easy to convince him like, wow, this is great.”

(ACS 15)

Mid-level hospital leader champions also were particularly important to sustainability. Most participants indicated that having a hospital leader—more so a department or program leader than an executive—as an ongoing, dependable champion was critical to sustainment of the ACS.

“I think a handful of our good experiences have been when hospital leadership have been on clinical services. For example, the CMO also is a hospitalist. When the CMO was covering the hospital service and utilized our services, then we got good positive feedback of, oh, that they could see in action kind of like how the service works and that it is encouraging. Hospital leadership did like a call for positive things or shout outs or something like a couple of months ago and a couple of people specifically mentioned the Addiction Consult Service as a great resource for patients. Which sort of brought it to the attention of hospital leadership in a positive light.”

(ACS 03)

A couple of participants who discussed the need for champions also pointed out that, while they are critically important, ACSs cannot rely on a single person to sustain ACSs, and that strategies are needed to ensure that ACSs are stable.

“Yeah, it’s basically our Division Chief is the reason we have the service and the reason that it’s been able to continue have support from high up but not that high up. She negotiates with our department Chair, I’m sure, who then makes sure that our dean knows that it’s a priority to keep this service. But I don’t take it for granted that our Division Chief will always be around. I have no idea, can’t tell the future. And so I don’t want to have the service basically only exist because one person in power is keeping it afloat. And I’m concerned that that’s what’s happening. So I’m always working towards, like, okay, well, how can we become indispensable?”

(ACS 11)

#### PSAT Domain: Funding Stability

**Figure 3:**
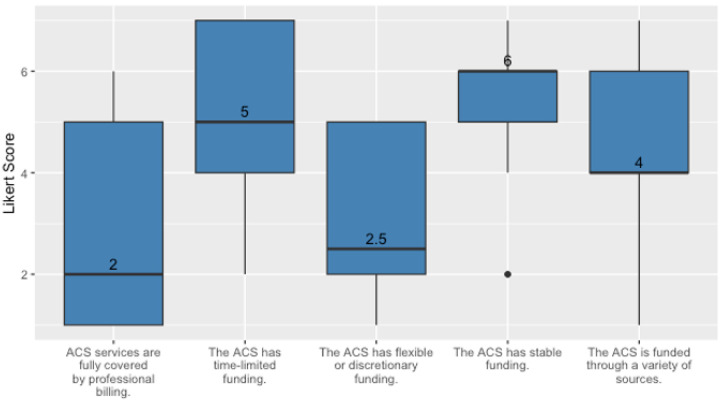
Median and IQR results for survey items assessing funding stability.

Survey results showed that startup resources in the form of grants or departmental support were needed to launch ACSs at most hospitals. Over time, hospitals took on blended funding models, with support from different sources across the hospital, however, most models are still thought to be unstable, with little full coverage for professional billing, and little flexible or discretionary funding. However, despite indications of inadequate billing coverage for the ACS, participants did believe that their ACS funding was generally stable (see Figure 3).

#### Theme: Startup grants were ideal for ACS startup, but other approaches were also used for initiating ACSs.

Regarding initial funding, interviewees described a range of start-up approaches, but most started with a grant or other type of support to get the ACS started. Sources of funding included state or Medicaid grants, federal grants (e.g., SAMHSA), and in some cases, institutional or departmental support. Some interviewees sought out grants to facilitate piloting or ramping up ACS services before trying to transition to relatively stable funding sources such as hospital support, partnerships with university departments, and billing for clinical services.

Despite the clear need for ACSs, a persistent challenge faced by nearly all the ACSs was assuring consistent financial support. From a purely economic standpoint, ACSs rarely generate profit and, in some instances, incur additional costs. This is partly because key team members—such as social workers and peer support specialists—are often unable to bill for their services, and because patients with OUD initiated on MOUD may experience longer hospital stays. However, interviewees described how those costs must be weighed against a range of metrics that indicate value, including lower readmission rates, improved patient health, and lower mortality rates.

“And it’s supported by their service line because we are doing these scheduled withdrawal management patients, and that ability to leverage some beds on our inpatient psych unit has really helped us with sustainability. I always worry that we’re gonna get the FTE revoked. I think that my repeated pitch in terms of what we’re doing here is just the extraordinary morbidity and mortality associated with Fentanyl use and really trying to advocate this service be a necessary service as opposed to a nice thing to have for hospitals. And it’s quite remarkable that [how] 25% of our patients in this hospital at least have substance use as at least one of their top three medical diagnosis coming into a hospital. And simultaneously, it was only 5 years ago that we were able to actually start doing this.”

(ACS 20)

#### Theme: Varied, blended funding models were used to sustain ACS, but funding was insufficient.

Ongoing funding for ACSs varied widely. In most programs, physicians billed for their professional services, while other ACS team members—such as social workers, nurses, and peer support or navigation specialists—do not bill for services. While peer support and social work services were billable in some states, actual collection rates were low, and many hospitals did not actively pursue this, contributing to a significant gap in funding for the roles required by an ACS.

“Well, so the medical staff, meaning the Attending and the Nurse Practitioner, are funded by the division, were hired through the division. The social workers are actually given to us by the hospital, which is really remarkable. And then the Peer Navigator joins us because she’s funded through a grant. And so I don’t know how long that funding is around for, but as long as I’ve been here, we’ve had a Peer through that funding source.”

(ACS 11)

#### PSAT Domain: Strategic Planning, Organizational Capacity

**Figure 4:**
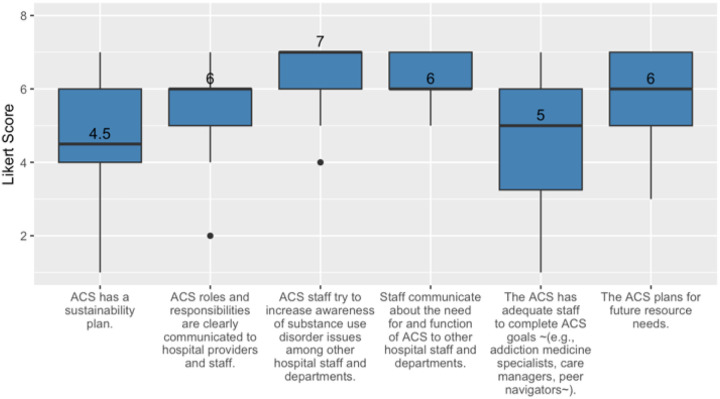
Median and IQR results for survey items assessing strategic planning and organizational capacity.

Survey responses illustrated strong agreement with statements about the existence of clear roles and responsibilities, and the need to increase awareness of SUD within the hospital, communicating about the need of the ACS, and to plan for future resource needs. Responses showed less agreement with statements about having a sustainability plan and adequate staffing for the ACS (see Figure 4).

#### Theme: Fostering and maintaining cross-disciplinary relationships throughout the hospital helped expand and sustain the ACS

Nurturing cross-departmental partnerships throughout the hospital was a key theme among participants, with participants discussing ongoing relationships with a diverse range of departments, including pharmacy, social work, psychiatry, cardiology, and transplant services (for ACSs that treat patients with alcohol use disorder). Participants noted that these relationships not only expanded the reach of the ACS to help more patients, but they also contribute to making the ACS an indispensable, highly utilized hospital service.

“So pharmacy is a huge partner and I love them. I think if I’ve learned anything over the years, anything can get done if you have a key champion that pushes enough. So having partners in different parts like pharmacy, nursing, social work, medicine, just knowing who’s your person, if you hit a wall is really important.”

(ACS 06)

#### Theme: Having an interprofessional staffing mix and cross-departmental participation increased capacity to sustain the ACS.

Having a mix of staff and embedding positions in departments throughout the hospital aided sustainability. Many ACSs used a cross-departmental approach. This included collaboration among different departments, including social work and psychiatry. Literature also shows a wide range of ACS models including staffing plans that draw from psychiatry, internal medicine and social work [8, 11].

“So, I’ll just say for sustaining, we had big structures for close support and supervision of the peers from the beginning. I embedded in the Department of Social Work that helps with retention and for them to be effective at their jobs. And so, that then led to sustaining the positions after adding to them and then sustaining even after the grants ended. In addition to the other key thing was to be able to demonstrate financial value to the hospital. So, being able to both monitor and show in particular readmissions in a hard sense and then also the fact that I think we’ve generally built good relationships, are helping patients, are a responsive service.”

(ACS 07)

To further support sustainability, several hospitals rotated fellows and trainees through the ACS to increase capacity and provide training.

“And so there was a lot of interest to bring in trainees to help with the clinical work, but also to provide education. That was like a really big thing. So we had no trainees in the service really and so we kind of expanded a lot, which has been wonderful.”

(ACS 02)

#### PSAT Domain: Monitoring and Evaluation

**Figure 5:**
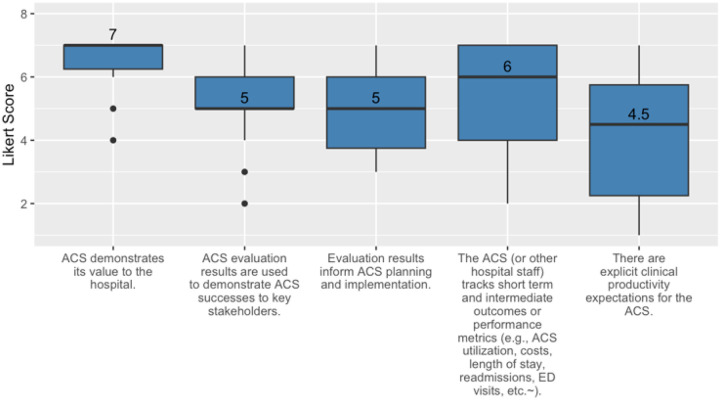
Median and IQR results for survey items assessing monitoring and evaluation.

Most participants indicated strong agreement with the statements describing that the ACS demonstrates value to the hospital and that the ACS tracks outcomes or performance metrics. Agreement was also high with statements about using evaluation results to demonstrate ACS success, that evaluation results inform planning, and that there are explicit productivity expectations for the ACS. There was a wide range of agreement with a statement on explicit clinical productivity expectations (see Figure 5).

#### Theme: Monitoring and evaluating the ACS were considered crucial to sustainment, but for most ACSs monitoring with standardized metrics was not required by the hospital; many had created their own dashboards to monitor progress and quality.

Although most ACSs were not required by hospitals to monitor their ACS using standardized metrics, many ACS clinicians and leaders created metrics and dashboards to monitor performance. Respondents indicated that monitoring and sharing results with administrative and departmental leadership was critical both for sustaining the ACSs and for evaluating and improving clinical processes. Metrics selected by ACSs varied, including but not limited to number of consultations, utilization and productivity of different ACS staff roles (e.g., physicians, social workers, peers), receipt of evidence-based treatment (i.e., MOUD), and provider and patient satisfaction.

“So I would create those dashboards every month after the ACS was implemented. And then we would just present them in monthly meetings with the team, the addiction consult team leads generally and then sometimes their admin. So like someone from behavioral health in the hospital, like whoever, like the addiction consult service was supervised by. And so we would kind of show them like, oh, yeah, you’re doing a great job in this. But like, what happened here? Why didn’t these people get medication? And it was more of just like a conversation for them to kind of think it through and figure out their workflows. So that was more from like, again, it was like me randomly doing it. I don’t even know if they like, one was like, you must do this. Just like something I did in my job, I like to do that stuff. So I did it.”

(ACS 15)

#### Theme: Metrics varied across hospitals

Some participants said they had specific targets for demonstrating value of the ACS to the hospital, with a focus on re-admission. Others noted that the key metric to share with hospital administrators to support ACS sustainment was staffing and productivity. Several participants said that length of stay was not a useful metric to report because it often increases for patients that receive services from the ACS.

“And then we could start to show outcomes that help the hospital like re-admission reduction, decrease in patient directed discharge. We are not focusing on length of stay. We probably lengthen length of stay in order to have a better connection to care. We were able to show and then in the sustaining phase, we have been able to show that those key outcomes that the hospital cares about in particular readmissions.”

(ACS 07)

“So, the service is funded and every few years we have to talk to the administration again about why they should keep funding our service. Because in some ways, we tried to look at this, we don’t even know if we help cut down on length of stay. Because part of what we do is help people stay here to get their infections treated or whatever it is when they otherwise would be leaving early AMA [against medical advice]. So, it’s complicated when you’re trying to show the value of a consultation service. Because everyone wants to look at length of stay and that sometimes isn’t actually what you’re helping.”

(ACS 22)

#### PSAT Domain: External Partnerships

**Figure 6:**
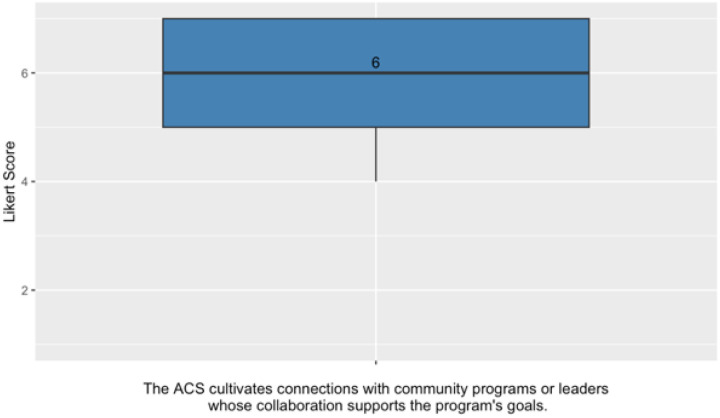
Median and IQR results for PSAT domain items assessing external partnerships.

Survey responses indicated strong agreement with the statement that the ACS cultivates connections with community programs or leaders whose collaboration supports the program’s goals, with a median score of 6 and an interquartile range of 5 to 7 (see Figure 6).

#### Theme: Relationships with community partners outside of the hospital were essential to ensuring linkage from the hospital to ongoing care, and to ACS sustainment.

A key element of success of ACSs described by participants was having frequent contact and strong relationships outside of the hospital with community partners, including treatment and harm reduction programs as well as with skilled nursing facilities (SNFs). Some participants noted that connections with SNFs were critical; without these relationships, patients who initiate MOUD in the hospital might be turned away from a SNF. These relationships could facilitate and often fast track patient referrals after discharge. Participants discussed how these relationships were key to successful sustainment; some participants noted that they included community partners in regular ACS meetings.

“[THIS HOSPITAL] specifically, I think the thing that’s really important, the number one facilitator that we have besides amazing staff, is our strong outpatient programs that we can link people to. And so we spend a lot of time and energy cultivating that relationship. But I think that that is really if you don’t have that you can’t do anything. So I think that’s a strength that we’ve had from day one.”

(ACS 08)

“But the leaders of those [COMMUNITY] programs which again are like in house staff would often come to our meetings as well, we would involve them. And that was partly also because we were trying to set up this bridge clinic and like using their space and their like infrastructure to have the bridge clinic established. And we wanted to make sure we got expedited into those programs. So they would set up fast tracking kind of workflows and things like that from the Addiction Consult Service to the outpatient program.”

(ACS 15)

#### Ongoing Barriers to ACS Sustainment

Participants discussed several specific challenges that continue to pose substantial threats to ACS sustainability.

#### Theme: The fee-for-service funding model created ongoing challenges for a full ACS, especially outside of academic settings.

Funding beyond initial startup grants was a challenge for most interprofessional ACSs. Several participants discussed how the fee-for-service model used in most hospitals only covered prescribing clinician time, with all other components of the ACS, including oversight, coordination, monitoring, and other staff, such as social workers and peers, were left to be funded in other ways. A few staff discussed how addiction still was not a priority at many hospitals, and so funding beyond billable services was difficult to come by.

“I think the barriers are that, again, I think oftentimes we’re asked to make dollars and a fee-for-service model, which is not really how we show the most benefit. I mean, fee-for-service models are disproportionate don’t benefit preventative care as much. They disproportionately favor surgical interventions and things that we don’t do. So I think it’s a challenge for us in a fee-for-service model because again, a lot of what we do is more about preventing overutilization, preventing overdose and death, preventing rehospitalization. And so I think our business case fits a lot better in an ACO model business case, but that’s just not where the health care system is right now. And so we could definitely benefit from more of both of those staff. It’s just a much harder argument to get hospitals to expand staff who can’t generate billing because again, it’s all about fee-for-service.”

(ACS 08)

#### Theme: Lack of adequate methadone treatment services outside the hospital, and barriers to linking patients on MOUD to acute care and skilled nursing facilities (SNFs) impeded referral and linkage to post-discharge care.

A few participants discussed a dearth of methadone providers in communities surrounding the hospitals as a barrier to initiating proper treatment in the hospital through the ACS and linking patients to care after discharge. Linkage to sub-acute rehabs and SNFs is also a challenge for people who start an MOUD in the hospital, because SNFs often do not take patients on methadone or other MOUDs.

“I think the biggest problem patients with this are like patients we start on methadone that are going to nursing homes or going to sub-acute rehab. The biggest barrier is getting those people treatment like the nursing homes. Like don’t want to take anyone who has any history of addictions even if it was remote. They also don’t want to—rehabs, sub-acute rehabs—don’t want to take them either. And so, then you have this problem of the non-ambulatory patient who needs, some kind of treatment who can’t get to a methadone clinic. The methadone clinics here have opened up a lot. But still, you have a homebound person who’s not going to be able to go and it’s really hard.”

(ACS 01)

#### Theme: Friction between hospital departments could impede ACS service delivery.

Almost a third of participants noted tension between medical and psychiatry departments as a challenge to ACS sustainability. For some, the tension stems from disagreement over where the ACS belongs, with some believing firmly that the ACS should be housed in a medical or hospitalist department and others feeling it belongs in psychiatry. Some of the conflicts were rooted in differences over whether substance use disorders should be treated in the hospital versus after discharge.

“However, I think the challenge really is, we’re largely an addiction psychiatry-based service. But the reality is, it turns out addiction psychiatrists are not as keen on doing consults. Addiction medicine folks, part of their training and part of their culture is to do inpatient work. And so, there’s a disproportionate number of addiction medicine folks looking to do consults, compared to addiction psychiatrists. So just from that alone, most psychiatrists don’t do consults, and most addiction psychiatrists don’t do consulting. So the reason why we are considering the option of reverting back to a separate service, is to actually stop it with addiction medicine physicians, and create a separate system. Because we still live in a very siloed world, and it’s actually a little tricky operationally to have two disciplines on the same service.”

(ACS 13)

“I would say it took us longer than I anticipated to get support and collaboration with Psychiatry. That was a barrier because they had some powerful influence and didn’t see us as allies. They saw us as competitors for some reason, even though we weren’t trying to compete with them. And they didn’t want to do the work, and they weren’t doing it, but I think they saw it as a reflection that they weren’t doing something that maybe they should have been doing… it was a difference of opinion of how you should treat substance use disorder in the hospital, and we believe that you should treat it. And so the philosophy of care was just different.”

(ACS 05)

#### Theme: Workforce issues such as finding staff to cover the ACS and staff turnover were a challenge for some ACSs.

Several participants noted that staff retention could be challenging for ACSs, particularly among advanced practice providers (APPs) who are newer to the field or lack prior experience working with patients with OUD. Additionally, some APPs and peer support specialists sought opportunities for career advancement but found limited pathways for promotion within the ACS. Leadership turnover and limited familiarity with addiction services among hospital administrators could further disrupt program continuity.

“I think another challenge is just around retention of staff. I have a lot of, again now long and high performing staff and looking for pathways to promotion. In our current medical system, there’s a lot less pathways to promotion for APPs versus physicians. Similarly, in terms of pathways to promotion for the peer recovery coach workforce, it’s a newer kind of workforce. I think there’s not as much understanding or value for that role in our current health care system. So a lot of the people who work for me are more nontraditional in the health care system. And so I think pathways to promotion and support. And similarly, I hire people with lived experience of using substances. And so that’s another thing that I think our health care system has not quite acclimated to is how do we best support people? And seeing their recovery, how would they define that? And so I think there’s a few unique layers, my colleagues in my same department don’t face.”

(ACS 08)

[1] We do not report the extent to which ACSs in our sample overlap (or do not overlap) with ACS samples from other articles, including Englander et al. (2022) in order to protect the confidentiality of participants.

## Discussion

While results from the START RCT have demonstrated the effectiveness of ACSs in increasing initiation of medications for OUD and improving linkage to treatment after discharge, and other studies have also demonstrated effectiveness of ACSs on these and other critical patient outcomes, less is known about the organizational and contextual factors that enable ACSs to be successfully launched and sustained within hospitals [5–7]. Our mixed-methods analysis addresses this gap by identifying key facilitators of and barriers to ACS implementation and sustainment.

Our findings highlight the importance of environmental support, including stakeholder buy-in, leadership endorsement, and broad recognition of the clinical need for OUD care, as a foundational condition for success. Internal champions, particularly those in mid-level leadership roles, are pivotal for building coalitions, securing resources, and aligning ACS initiatives with hospital priorities. While startup funding often comes from soft funds, successful ACSs tend to evolve toward a blended funding model, with many programs relying on staffing or indirect subsidization by teaching services. Cross-departmental integration and interprofessional staffing are thought to be essential to sustainability for expanding the ACSs’ reach and embedding their practices into existing hospital workflows.

Although evaluation practices differed across hospitals, many ACSs use performance monitoring to strategically demonstrate their value, such as tracking readmission rates for patients who were seen by the ACS and sharing these data with leadership to justify continued investment. External partnerships with outpatient programs and community organizations are also critical for enabling successful discharge plans and reinforcing the ACS’s role within a broader continuum of care.

Persistent barriers to ACS sustainability include fee-for-service reimbursement shortfalls, lack of access to methadone services in many communities, difficulties transferring patients on MOUD to SNFs, interdepartmental coordination challenges, and workforce retention issues. Despite relatively high PSAT domain scores, indicating strong internal support for sustainment, many ACSs continue to struggle with financial vulnerability. Funding stability is the lowest scored factor for ACS sustainability. Echoing findings from Priest et al., interviewees described a fundamental misalignment between ACS functions and prevailing reimbursement models that primarily support prescribers but often exclude other essential team members, such as social workers and peer specialists [12]. Finally, findings caution against overreliance on individual champions, emphasizing instead the need for institutional structures and policies to promote long-term program stability beyond the tenure of specific leaders.

In Table 6, we summarize key takeaway lessons that practitioners (e.g., those involved with starting or sustaining an ACS) can use, or be aware of, throughout the development and evolution of their ACS. While each ACS ultimately needs to be tailored to its local environment, these general takeaways can be informative for those operating in a wide range of hospital environments.

This study faces some limitations including the fact that the ACSs represented in this study tend to be relatively well-resourced hospitals, many with secondary affiliations (e.g., with universities or with the VA) and many in urban areas. Priest et al. noted a similar limitation in their 2020 study [12]. It seems likely that well-resourced ACSs are more likely to be initiated, sustained, and better known to those in the addiction medicine field. Future research can explore the extent to which less well-resourced hospitals have tried or plan to try implementing ACSs. Additionally, future research can explore cases in which ACSs were initiated but not sustained over time. Finally, while there was some variation in the taxonomic category of the ACSs in this study, interview themes tended to be consistent across participants. Further research can build on Englander et al.’s [11] call to explore outcomes of different types of ACS models by exploring nuances in implementation and sustainability in relation to ACS outcomes.

## Conclusion

Our analysis identified multiple domains relative to ACS initiation and sustainability, including environmental support, funding stability, organizational capacity, evaluation, and external partnerships. Prevailing themes include the importance of stakeholder buy-in, leadership support, and champions at different levels; the use of blended funding sources; the importance of cross-departmental collaboration; the value of metrics to monitor ACSs; and the role of community linkages in sustaining ACSs. By using PSAT domains to inform our analysis, we have developed a framework that other providers and administrators can use to plan, implement, and support an ACS over time.

## Supplementary Material

Supplementary Files

This is a list of supplementary files associated with this preprint. Click to download.

- Table2InterviewCodebook.docx
- Table3ParticipantDemographics.docx
- Table4CharacteristicsofACSs.docx

Tables

Tables 2, 3, and 4 are available in the Supplementary Files section.

## Figures and Tables

**Table 1.** Interview Guide

PSAT category	Interview Questions
Environmental support	Who were the key decision-makers at [HOSPITAL] who influenced whether the ACS was adopted and sustained? What partnerships within the hospital have been critical to implementing and sustaining the ACS?
Funding stability	How did funding work during the initiation and development of the ACS? How does funding work for sustaining the ACS? Does the ACS bill for its services? What mechanisms or sources fund this program? How is funding sustained? What have been the biggest funding barriers? How have these been overcome?
Organizational capacity	You told us previously […]. Were these the roles you started with? If not, how have the roles changed and why? Has the presence of the ACS altered or changed other clinical processes or services?
Strategic planning	How does strategic planning for new programs typically occur at [HOSPITAL]? Who makes sure that planning for programs aligns with existing hospital goals? What implementation strategies are typically used to implement new practices? What do you think the most important factor was for implementing this ACS?
Communications	Tell me about how you shared information about the ACS in the hospital when it was first implemented? How does communication with those hospital stakeholders work today? To what extent have community stakeholders been informed about the ACS?
Monitoring and Evaluation	How is the ACS monitored and evaluated? Who oversees monitoring and evaluation? What metrics or goals does the ACS report on? Has the ACS evolved over time? Is the ACS regularly adapted to meet specific objectives? With whom do you communicate successes related to the ACS?
External Partnerships	What partnerships outside of the hospital have been critical to implementing and sustaining the ACS? What has been the role of these partners in implementing the ACS?
Barriers	What do you think were the most significant barriers to implementing and sustaining the ACS at [HOSPITAL]? Do any of these barriers still exist? Can you discuss any barriers impacting the ACS now?

**Table 5.** Summary of Themes from Qualitative Interviews

PSAT Domain	Theme
Environmental Support (Including internal partnerships)	Widespread stakeholder buy-in for initiating the ACS was common at most hospitals due to consensus around the need for OUD treatment among inpatients.
	Leadership support and having “champions” throughout the hospital have been critical to both the initiation and sustainment of ACSs.
Funding stability	Startup grants are ideal for ACS startup, but other approaches were also used for initiating ACSs.
	Varied, blended funding models are used to sustain ACS, but funding isn’t stable.
Strategic Planning, Organizational Capacity	Fostering and maintaining cross-departmental relationships throughout the hospital can help expand and sustain the ACS
	Having an interprofessional staffing mix and cross-departmental participation can increase capacity to sustain the ACS.
Monitoring and Evaluation	Monitoring and evaluating the ACS are considered crucial to sustainment, but for most ACSs monitoring with standardized metrics is not required by the hospital. Despite the lack of required metrics, many ACSs have created their own dashboards to monitor progress and quality.
	Metrics varied across hospitals.
External Partnerships	Relationships with community partners outside of the hospital are essential to ensuring linkage from the hospital to ongoing care, and to ACS sustainment.
Ongoing Barriers to ACS Sustainment	The fee-for-service funding model creates ongoing challenges for a full ACS, especially outside of academic settings.
	Lack of adequate methadone treatment services outside the hospital and barriers to linking patients on MOUD to acute care and skilled nursing facilities (SNFs) impedes referral and linkage to post-discharge care.
	Friction between hospital departments can impede ACS service delivery.

**Table 6:** Summary of Takeaways Related to ACS Sustainment

PSAT Domain	Key Takeaways for Practitioners
Environmental Support (Including internal partnerships)	Facilitator:
	Stakeholder buy-in
	Broad recognition of the clinical need for OUD
	Leadership support
	Champions throughout the hospital
Funding Stability	Facilitator:
	Grant or other support for start-up phase
	Transition to a stable funding source including internal funding
	Barrier:
	ACSs typically do not earn a profit due to key staff members who cannot bill
Strategic Planning, Organizational Capacity	Facilitator:
	Cross-disciplinary relationships within the hospital
	Interprofessional staffing mix and cross-departmental participation
Monitoring and Evaluation	Facilitator:
	Monitoring and evaluating the ACS are considered crucial to sustainment
	Barrier:
	Metrics are not standardized or formalized within hospitals or across sites
External Partnerships	Facilitator:
	Relationships with community partners help ensure linkage from the hospital to ongoing care
Ongoing Barriers to ACS Sustainment	Additional barriers:
	Fee-for-service funding model is challenging, especially outside of academic settings
	Lack of adequate methadone treatment services outside the hospital, and barriers to linking patients on MOUD to acute care and skilled nursing facilities (SNFs), impede referral and linkage to post-discharge care.
	Friction between hospital departments can impede ACS service delivery
	Workforce issues, such as finding staff to cover the ACS and staff turnover, are challenges for some ACSs

## Data Availability

Data from the survey and deidentified qualitative data generated and analyzed during this study can be made available from the corresponding author on reasonable request and with execution of appropriate Data Use Agreements.

## Abbreviations

ACS: Addiction Consult Service / Addiction Consultation Service
AMA: Against Medical Advice
APP: Advanced Practice Provider
CI: Confidence Interval
CMO: Chief Medical Officer
FTE: Full-Time Equivalent
IQR: Interquartile Range
IRR: Inter-Rater Reliability
MOUD: Medication for Opioid Use Disorder
OUD: Opioid Use Disorder
PSAT: Program Sustainability Assessment Tool
RCT: Randomized Clinical Trial
SNF: Skilled Nursing Facility
START: Substance Use Treatment and Recovery Team
SUD: Substance Use Disorder
U.S.: United States
VA: Veterans Affairs
